# HDAC Inhibitors Act with 5-aza-2′-Deoxycytidine to Inhibit Cell Proliferation by Suppressing Removal of Incorporated Abases in Lung Cancer Cells

**DOI:** 10.1371/journal.pone.0002445

**Published:** 2008-06-18

**Authors:** Guolin Chai, Lian Li, Wen Zhou, Lipeng Wu, Ying Zhao, Donglai Wang, Shaoli Lu, Yu Yu, Haiying Wang, Michael A. McNutt, Ye-Guang Hu, Yingqi Chen, Yang Yang, Xin Wu, Gregory A. Otterson, Wei-Guo Zhu

**Affiliations:** 1 Department of Biochemistry and Molecular Biology, Peking University Health Science Center, Beijing, China; 2 Department of Pathology, Peking University Health Science Center, Beijing, China; 3 State Key Laboratory of Molecular Biology, Institute of Biochemistry and Cell Biology, Chinese Academy of Sciences, Shanghai, China; 4 Department of Internal Medicine, Division of Hematology Oncology, Comprehensive Cancer Center, Ohio State University, Columbus, Ohio, United States of America; University of Hong Kong, China

## Abstract

5-aza-2′-deoxycytidine (5-aza-CdR) is used extensively as a demethylating agent and acts in concert with histone deacetylase inhibitors (HDACI) to induce apoptosis or inhibition of cell proliferation in human cancer cells. Whether the action of 5-aza-CdR in this synergistic effect results from demethylation by this agent is not yet clear. In this study we found that inhibition of cell proliferation was not observed when cells with knockdown of DNA methyltransferase 1 (DNMT1), or double knock down of DNMT1-DNMT3A or DNMT1-DNMT3B were treated with HDACI, implying that the demethylating function of 5-aza-CdR may be not involved in this synergistic effect. Further study showed that there was a causal relationship between 5-aza-CdR induced DNA damage and the amount of [^3^H]-5-aza-CdR incorporated in DNA. However, incorporated [^3^H]-5-aza-CdR gradually decreased when cells were incubated in [^3^H]-5-aza-CdR free medium, indicating that 5-aza-CdR, which is an abnormal base, may be excluded by the cell repair system. It was of interest that HDACI significantly postponed the removal of the incorporated [^3^H]-5-aza-CdR from DNA. Moreover, HDAC inhibitor showed selective synergy with nucleoside analog-induced DNA damage to inhibit cell proliferation, but showed no such effect with other DNA damage stresses such as γ-ray and UV, etoposide or cisplatin. This study demonstrates that HDACI synergistically inhibits cell proliferation with nucleoside analogs by suppressing removal of incorporated harmful nucleotide analogs from DNA.

## Introduction

It is well known that DNA methylation is associated with histone acetylation status in regulation of gene expression [1]–[4] or cell proliferation and aging [5]. This linkage between histone status and DNA methylation was well confirmed by Cameron *et al* who found that several genes silenced by methylation were reactivated when treated with demethylating agent 5-aza-CdR and histone deacetylase inhibitor (HDACI) trichostatin A (TSA) together, but were not reactivated in the presence of 5-aza-CdR or TSA alone [6]. Later, several laboratories including our own extended these findings to set up a therapeutic strategy for cancer treatment, in which 5-aza-CdR acts in conjunction with depsipeptide/TSA to induce significant apoptotic cell death [7]–[10]. Since DNA methylation in the promoter region is associated with HDAC1 by a methyl-binding protein MeCP2 [11], it is believable that certain genes, if hypermethylated in their promoter region, are more tightly packed by histones and thus transcription factors access their DNA binding sites only with greater difficulty. Consequently, cell death related genes, which are silenced due to hypermethylation, could be reactivated by treatment with 5-aza-CdR; this reactivation should be strengthened by HDACI, and at the same time, cell death should be more readily observable as well.

However, 5-aza-CdR also plays an anti-neoplastic role which is methylation-independent [12]–[14]. 5-aza-CdR may act directly on mitochondria and induce apoptosis in mammalian cells [14]. A direct methylation independent evidence for the 5-aza-CdR induced cell death is that 5-aza-CdR significantly enhances expression of Apaf-1 or p19^INK4d^ to induce cell death, however the promoter regions of these genes are totally unmethylated [12], [13]. 5-aza-CdR has also been reported to induce a synergistic effect by increasing the cisplatin bound to DNA, via a change in the topology of DNA brought about by 5-aza-CdR without its functioning as a demethylating agent [15]. These data suggest that 5-aza-CdR plays diverse roles in cells which include both demethylating functions and methylation-independent functions.

The cytotoxicity of 5-aza-CdR results from its capacity to damage DNA as 5-aza-CdR is a nucleoside analog (NA) and can be incorporated into the DNA backbone [16], [17], which in turn may induce formation of a covalent adduct between the 5-aza-CdR molecule and methyltranferases [16]. It has been demonstrated that NAs such as 5-aza-CdR or cytarabine (Ara-C), are phosphorylated into their triphosphate form and are then incorporated into DNA during replication [18]. Subsequently, the incorporated NA serve as an abase that may induce DNA damage, mutations and stalling of the DNA replication fork [19], [20]. These changes in the DNA backbone are harmful, and DNA damage sensors such as DNA-PK, p53, ATM and ATR recognize these damaged sites and repair the abnormal DNA [21], [22]. Previously, both our group and other researchers directly confirmed that 5-aza-CdR induces DNA damage and elicits p53-dependent biological reactions [23]–[25]. However, if the abnormal DNA changes induced by the incorporation of NAs are overwhelmed, or the DNA repair systems are severely inhibited, the cells will undergo apoptosis [26].

HDAC inhibitors are novel and effective anticancer agents [27], [28], which are involved in regulating many genes including activation of p53 [29], or downregulating anti-apoptotic genes [28], [30] to exert an anti-neoplastic effect. Therefore, investigation of whether or not HDACI also affects DNA repair enzymes to enhance NA-induced DNA damage is called for.

In this study, we confirm that 5-aza-CdR acts in concert with HDACI to induce a significant inhibition of cell proliferation in a methylation independent manner. 5-aza-CdR was incorporated into DNA in a dose and time dependent manner that was clearly associated with induction of DNA single-strand breaks (SSB) by 5-aza-CdR. HDAC inhibitors significantly suppressed removal of the incorporated 5-aza-CdR from DNA and thus produced a synergistic effect resulting in inhibition of cell proliferation

## Materials and Methods

### Cell culture and chemical treatment

Human lung cancer cell lines A549 and H719 were used in this study. Both 5-aza-CdR (dissolved in 50% acetic acid) and Ara-C (dissolved in DMSO) (both purchased from Sigma, St Louis, MO) were added fresh into the medium every 24 hrs. TSA (Sigma) was dissolved in ethanol. Depsipeptide (kindly provided by the NIH) was dissolved in DMSO.

### Determination of cell proliferation by MTT assay

Equal numbers of cells (approximately 5000/well) were seeded into a 96-well plate 24 hrs prior to experimentation. Cells were treated with 5-aza-CdR, Ara-C, γ-irradiation and HDACI alone or in combination. After treatment, MTT dye solution (Sigma) was added into the 96-well plate. The corrected absorbance of each sample was calculated by comparison with the untreated control.

### RT-PCR

To determine if knockdown of DNA methyltransferases (DNMTs) is effective in DNA demethylating and reactivating silenced genes, RT-PCR was performed to detect changes in expression of p21 that is silenced due to a hypermethylation in the *p21* promoter [31]. The PCR primers were as follows: p21, left primer- GGA AGA CCA TGT GGA CCT GT and right primer- GGA TTA GGG CTT CCT CTT GG; *β*-actin, left primer- TGG AGA AGA GCT ACG AGC TGC CTG and right primer- GTG CCG CCA GAC AGC ACT GTG TTG.

### Western Blot

To identify changes in protein expression in cells treated with knockdown of DNMTs or 5-aza-CdR, cells were harvested with a scraper and then washed once with cold phosphate-buffered saline. The cells were then lysed in lysis buffer (50 mM Tris-HCl, 250 mM NaCl, 5 mM EDTA, 50 mM NaF, 0.15% Igepal CA-630, and 1.5 mM phenylmethylsulfonyl fluoride). Equal amounts of proteins (100–150 µg) were size fractionated on 7.5–12.5% SDS-PAGE. In addition, to determine the amount of the chromatin-bound RPA, cells were lysed in solution A (50 mM Tris-HCl, pH 7.8, 420 mM NaCl, 1 mM EDTA, 0.5% NP-40, 0.34 M sucrose, 10% glycerol, 1 mM Na_3_VO_4_ and protease inhibitor cocktail). To obtain nuclear extracts, cells were lysed in buffer B (10 mM HEPES, pH 7.9, 10 mM KCl, 1.5 mM MgCl_2_, 0.34 M sucrose, 10% glycerol, 0.1% Triton X-100, protease inhibitor cocktail). Isolated nuclei were washed once with buffer B and further lysed in buffer A as above. To obtain chromatin-bound proteins, isolated nuclei were lysed in buffer C (3 mM EDTA, 0.2 mM EGTA, 1 mM DTT) and further lysed in buffer A as above.

The antibodies used were anti-PARP (Santa Cruz, F-2), anti-RPA (Santa Cruz, MA34 and MA70-2), anti-γ-H2AX (Upstate, 05-636), anti-DNMT1(Santa Cruz, H-300), anti-DNMT3A (Santa Cruz, P-16), anti-DNMT3B (a gift from Dr. Xu, G, Shanghai Institute of Biological Science) and β-actin antibodies were purchased from Santa Cruz.

### DNA bisulfite treatment and bisulfite sequencing

To evaluate changes in DNA methylation status after treatment with knockdown of DNMTs, DNA was treated with sodium bisulfite and purified for PCR as described previously [31]. The primers for sequencing the *p21* promoter were as follows: left primer, 5′-GGG AGG AGG GAA GTG TTT TT -3′ and right primer, 5′-ACA ACT ACT CAC ACC TCA ACT-3′. The PCR products were gel extracted (Qiagen, Valencia, CA) and ligated into a pGEM-T easy vector, by using the TA cloning system (Promega, Madison, WI). Transformed bacteria DH5α were cultured overnight, and the plasmid DNA was isolated using a kit (Qiagen). At least 10 separate clones were chosen for sequence analysis.

### Comet assay

The comet assay was performed as described previously [24]. In brief, frosted microscopic slides were covered with 0.5% normal melting agarose at 60°C. About 10^5^ 5-aza-CdR treated or untreated cells in PBS were mixed with an equal amount of 1% low melting agarose to form a cell suspension. After electrophoresis, slides were examined at 600× magnification and pictures were taken with a fluorescence microscope (TCS, Leica, Manheim, Germany).

### Analysis of exclusion of incorporated [^3^H]-5-aza-CdR from DNA

The radiolabeled nucleoside analog incorporation assay was performed as described previously with modifications [32]. [^3^H]-5-aza-CdR was purchased from Moravek Biochemicals (MT1676, 1 mCi/ml, Brea, CA). H719 and A549 cells were treated with [^3^H]-5-aza-CdR at 1 µM for 48 hrs with or without depsipeptide at 0.1 µM for the final 6 hrs (from 42 to 48 hrs) and the cells were then washed with cold PBS. The cells were then replaced in [^3^H]-5-aza-CdR free medium and incubated for 6, 12 or 24 hrs. Cold trichloroacetic acid (TCA, 50%) was then added to the cell solution and the TCA-insoluble DNA was placed on a glass microfiber filter (Whatman, Maidstone, England). This glass filter with TCA-insoluble DNA was placed in a vial to count radioactivity with a Beckman LS 5000 Liquid Scintillation Counter.

### Establishment of cell line with a stable DNMT1-deficient clone

The pCMVneo -DNMT1 antisense plasmid was kindly provided by Dr. SB Baylin [33]. The vector was transfected into H719 cells using the Qiagen Effectene Transfection kit (Qiagen, Hilden, Germany) according to the manufacturer's instructions. Twenty-four hrs after the first transfection, a second transfection was performed to strengthen the efficiency of the RNAi, and then 800 µg/ml of G418 was added to select stable cells with DNMT1 antisense.

### RNA interfering with DNMT3A, DNMT3B

The RNAi targeted oligonucleotide sequences are as follows: CAT CCA CTG TGA ATG ATA ATT (DNMT3A) and GAT CAA GCT CGC GAC TCT C (DNMT3B) (purchased from Shanghai GeneChem Company). The oligonucleotides for DNMT3A and DNMT3B siRNA were transfected into cells by using lipofectamine 2000 for 48 hrs.

## Results

### 5-aza-2′-deoxycytidine cooperates with HDACI to induce inhibition of cell proliferation by a methylation independent mechanism

**Figure 1:**
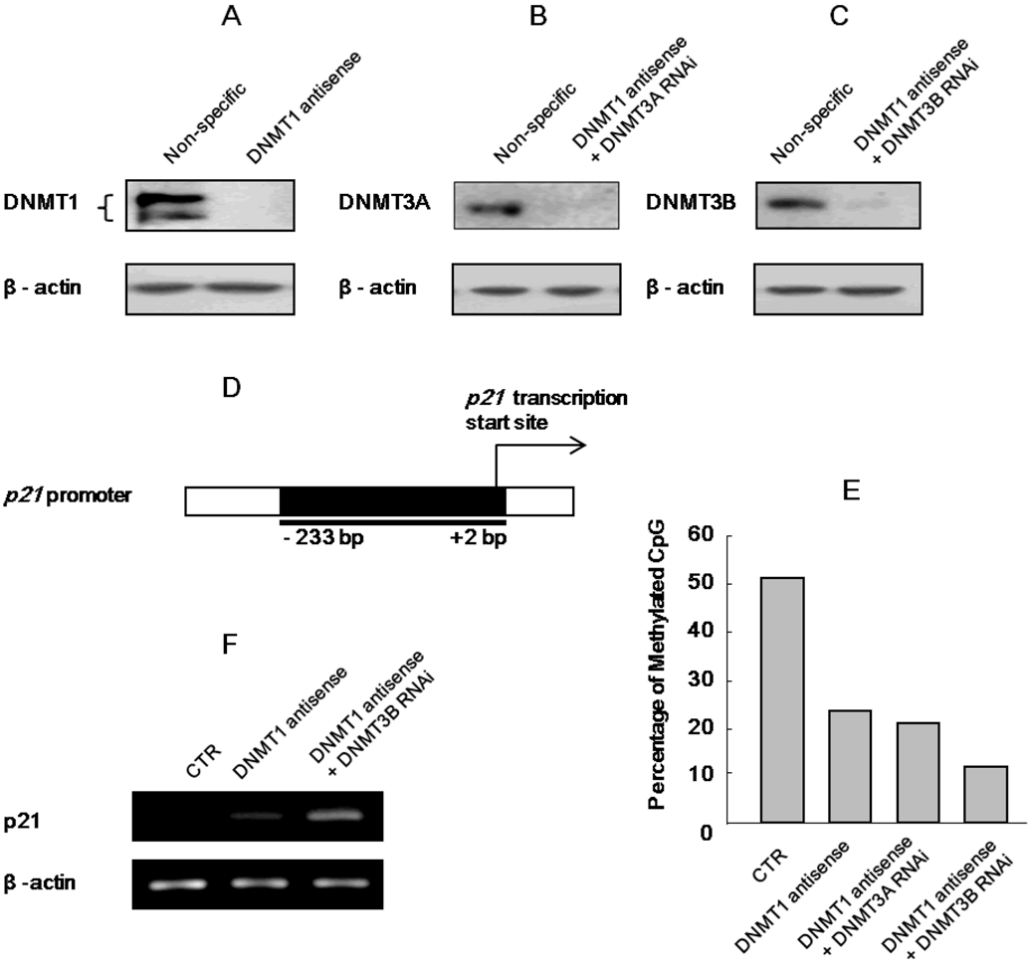
Suppression of DNA methyltransferases is sufficient to induce demethylation. (A) Representative Western blot indicates that DNMT1 protein level in H719 cells was significantly decreased in the DNMT1-deficient stable clone when compared to the non-specific oligonucleotide treated control. (B) and (C) Representative Western blots also show that DNMT3A or DNMT3B protein level was significantly decreased after RNAi treatment in DKD of DNMT1/DNMT3A cells or DKD of DNMT1/DNMT3B cells, respectively. β-actin is shown as a loading control in the lower panel. (D) A schematic diagram showing the *p21^Waf1/Cip1^* promoter, with the black region indicating the fragment which underwent bisulfite sequencing (−233 to +2 relative to transcription start site). (E) With bisulfite sequencing, 10 clones were randomly selected and the methylation status of the *p21^Waf1/Cip1^* promoter was determined in untreated H719 cells or in the H719 cells treated with knockdown of DNMT1, DKD of DNMT1/DNMT3A or DKD of DNMT1/DNMT3B. (F) RNA was extracted from H719 cells treated with DKD of DNMT1/DNMT3B, and RT-PCR was performed to detect expression of p21 mRNA. β-actin is shown as a loading control for the RT-PCR analysis.

It has been reported that 5-aza-CdR acts together with HDAC inhibitors to synergistically induce cell death or inhibition of cell proliferation [7]–[10]. To investigate whether the role of 5-aza-CdR involved in this synergistic inhibition of cell proliferation when combined with HDACI is epigenetic, two double knock down (DKD) clones (DKD of DNMT1/DNMT3A and DKD of DNMT1/DNMT3B) were established. To avoid inducing a difference in efficiency of transfection when cells were transfected with two vectors or two RNAi oligonucleotides, a DNMT1 deficient stable clone was first established by transfection of a vector with antisense DNMT1 cDNA into H719 cells and selected with G418. As shown in Fig. 1A, DNMT1 protein is well knocked down in this stable H719 cell line. Thereafter, DKD of DNMT1/DNMT3A, or DNMT1/DNMT3B was carried out by transfection of DNMT3A or DNMT3B RNA oligonucleotides into the DNMT1 knockdown stable cells. Figure 1B–C shows clearly that DNMT3A and DNMT3B protein levels were significantly reduced by these RNAi treatments. To evaluate whether these DKDs of DNMTs were efficient for demethylating DNA, the methylation status of the *p21^Waf1/Cip1^* promoter was chosen for testing with bisulfite sequencing because the *p21^Waf1/Cip1^* promoter is highly methylated in H719 cells [31]. Fig. 1D represents a schematic diagram showing the promoter region of *p21^Waf1/Cip1^*and the fragment designated for bisulfite sequencing. As shown in Fig. 1E, the *p21^Waf1/Cip1^* promoter in H719 cells was highly methylated (51.25% of CGs in the *p21^Waf1/Cip1^* promoter were methylated in 10 selected clones). However, methylated CGs were decreased to 23.75%, 21.5% and 12.5% when DNMT1,DNMT1/DNMT3A and DNMT1/DNMT3B were subjected to double knock down with 10 selected clones (Fig. 1E), indicating that knockdown of DNMT1 and DNMT3B together was the most efficient for DNA demethylation. This decrease in methylation of the *p21^Waf1/Cip1^* promoter was clearly associated with expression of p21 as measured with RT-PCR (Fig. 1F).

**Figure 2:**
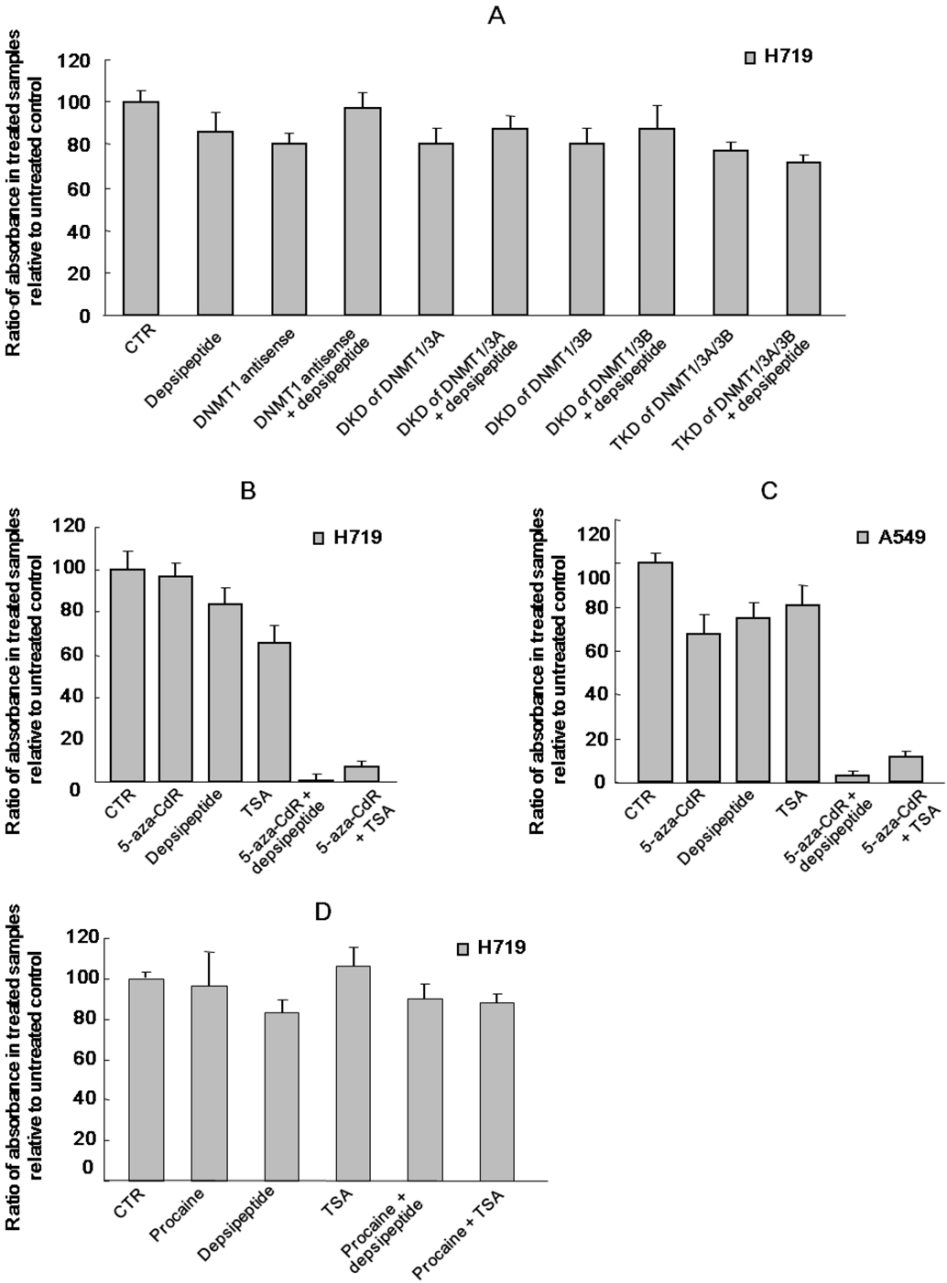
HDAC inhibitor depsipeptide or TSA and knockdown of DNMTs do not cooperate to inhibit cell proliferation. (A) H719 cells with deficient DNMT1, DKD of DNMT1/DNMT3A, DKD of DNMT1/DNMT3B, or TKD (triple knockdown) of DNMT1/DNMT3A/DNMT3B were treated with or without depsipeptide at 0.1 µM for 6 hrs, and cell proliferation was tested with the MTT assay. (B) and (C) 5-aza-CdR (1 µM for 72 hrs) cooperates with HDAC inhibitor depsipeptide (0.1 µM for the final 6 hrs) or TSA (1 µM for the final 6 hrs) to synergistically induce inhibition of cell proliferation in H719 (B) or A549 cells (C). (D) H719 cells were treated with procaine (0.5 mM) for 72 hrs, and depsipeptide (0.1 µM for the final 6 hrs) or TSA (1 µM for the final 6 hrs) was then added to the treated cells. All data are the results of two separate experiments.

An unexpected finding was that we did not observe a difference in cell proliferation when H719 cells with DKDs or TKDs (knockdown of DNMT1/3A/3B) were treated with or without depsipeptide and assayed with MTT (Fig. 2A). In contrast, cell proliferation was significantly decreased in both A549 and H719 cells which were treated with 5-aza-CdR and HDACI depsipeptide/TSA, although 5-aza-CdR alone and depsipeptide/TSA alone had little effect on inhibition of cell proliferation at the doses used (Fig. 2B–C). However, 5-aza-CdR-induced inhibition of cell proliferation was cell line dependent. For example, 5-aza-CdR showed an obvious effect in inhibiting proliferation of A549 cells (Fig. 2C), but had no effect in H719 cells (Fig. 2B). These differences in the 5-aza-CdR-induced effect on cell proliferation between A549 and H719 cells resulted from the p53 status of the treated cells [24]. A549 cells have a wild-type p53, whereas H719 cell p53 is mutated [31]. In addition, although procaine has been reported to be a demethylating agent [34], synergistic inhibition of cell proliferation was not observed when H719 cells were treated with procaine (0.5 mM for 72 hrs) and depsipeptide (0.1 µM for 6 hrs at 66–72 hrs) or TSA (1 µM for 6 hrs at 66–72 hrs) (Fig. 2D). These data suggest that the role of 5-aza-CdR in its synergistic effect with HDACI in inducing inhibition of cell proliferation may not result from its demethylating function.

### 5-aza-CdR induces cytotoxicity by incorporation into DNA as a nucleoside analog

**Figure 3:**
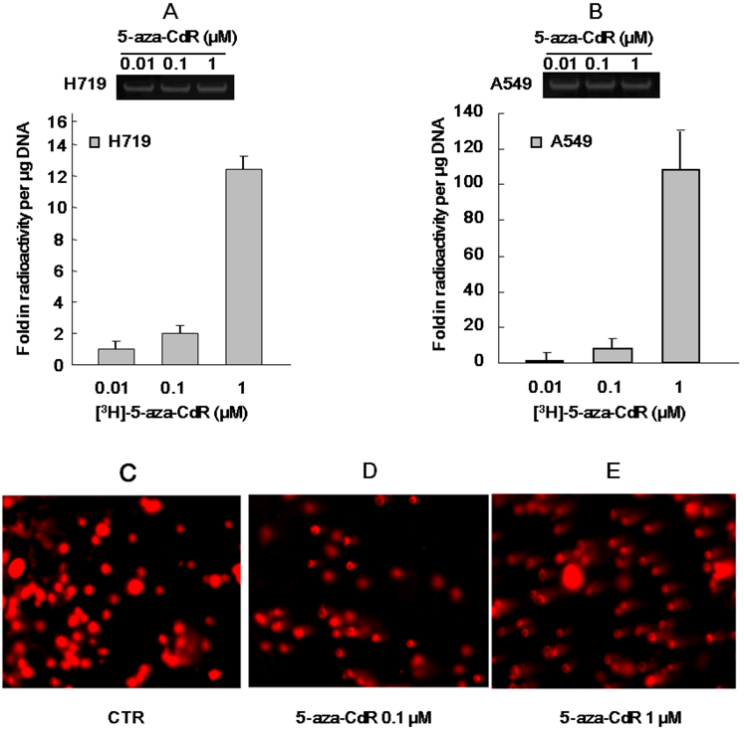
The amount of [^3^H]-5-aza-CdR incorporated into DNA parallels the degree of 5-aza-CdR induced DNA damage. (A) H719 or (B) A549 cells were incubated with [^3^H]-5-aza-CdR for 24 hrs at different concentrations and then washed with cold PBS. The cells were incubated in fresh medium for another 24 hrs, and DNA was then precipitated with cold TCA. Radioactivity was measured and relative radioactivity per µg was counted. Same amount of DNA was loaded and run at 1% of agarose gel at 100 V for 5 min as loading control, which is shown at upper panel of Fig. 3A and Fig. 3B, respectively. The results presented are from two separate experiments (mean±SD) DNA damage induced by 5-aza-CdR in H719 cells was determined by the comet assay. (C) Untreated control, (D) 5-aza-CdR at 0.1 µM for 72 hrs and (E) 5-aza-CdR at 1 µM for 72 hrs.

It is well known that 5-aza-CdR is phosphorylated to its triphosphate form in cells and then incorporated into DNA during replication [18]. In order to investigate the degree of 5-aza-CdR incorporation into cells, [^3^H]-5-aza-CdR was added to A549 or H719 cells for 24 hrs and DNA was then extracted with TCA precipitation. Radioactivity per µg DNA was measured. As shown in Fig. 3A–B, incorporation of [^3^H]-5-aza-CdR into DNA was concentration dependent in both cell lines. Incorporated radioactivity per µg DNA at 0.1 µM and 1 µM of [^3^H]-5-aza-CdR increased ∼2- and ∼12-fold respectively in H719 cells, and ∼10- and ∼110-fold respectively in A549 cells when compared to the radioactivity of 0.01 µM [^3^H]-5-aza-CdR in both cell lines.

There was a relationship between incorporated 5-aza-CdR and DNA damage as determined with the comet assay. Figure 3C–E shows that 5-aza-CdR exhibited a dose-dependent capacity for DNA damage in A549 cells, which was demonstrated by presence of a DNA tail. Larger DNA tail area and longer DNA tail length represent greater DNA damage. Table 1 shows a statistic analysis of the comet assay comparing 5-aza-CdR treated samples to an untreated control. This data parallels the incorporated amount of 5-aza-CdR, and implies that 5-aza-CdR plays a role in cytotoxicity by incorporation into DNA.

**Table 1:** DNA damage induced by 5-aza-CdR was detected with the comet assay.

Treatment	Tail area/Total area ( % )	DNA tail length
0 µM	11.3±8	4.1±0.2
0.1 µM	35.8±11	7.75±0.8
1 µM	52.8±6	15.5±0.6

With fluorescence microscope analysis software, the percentage of the DNA tail area was divided to total DNA area for each A549 cell, and percentage of DNA tail length divided to total DNA length are counted. The data represent an average from 50 cells with standard error.

**Figure 4:**
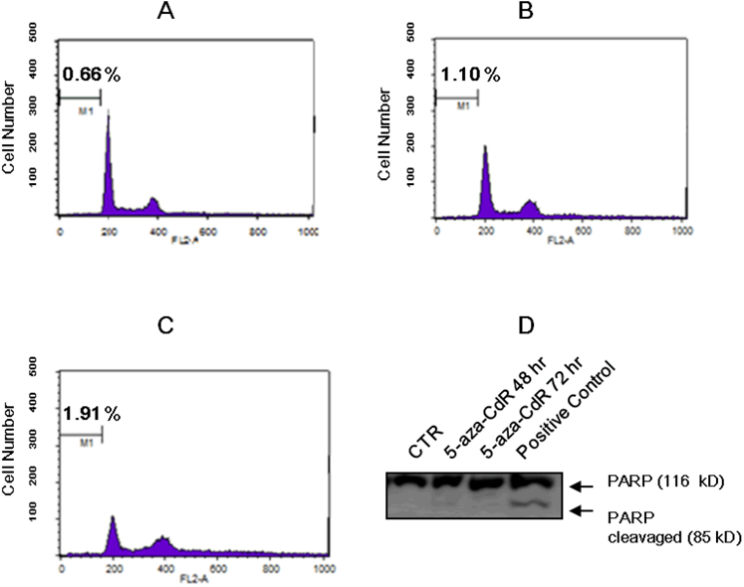
5-aza-CdR at limited dose does not induce apoptosis. (A–C) To determine whether 5-aza-CdR-induced DNA damage induces inhibition of cell proliferation via apoptotic cell death, a flow cytometry analysis was performed. Untreated A549 cells (A) or A549 cells were treated with 5-aza-CdR at 0.1 µM (B) or at 1 µM for 72 hrs (C), and the cells were then harvested for staining with Propidium Iodide (PI). DNA content with smaller than G_0_/G_1_ was considered as apoptotic DNA (area of M1). (D) A549 cells were treated with 5-aza-CdR at 1 µM for 48 or 72 hrs and protein was extracted for Western blotting using anti-PARP to detect PARP cleavage in cells treated with 5-aza-CdR. An apoptotic positive control was shown at the lane of the far right, in which there are two bands indicating the PARP (116 KD) and PARP cleavaged (85 KD).

As apoptosis can also give a positive result in the comet assay [35], we harvested 5-aza-CdR treated cells and performed flow cytometry analysis to determine whether 5-aza-CdR-induced DNA damage elicits apoptotic cell death. The cells with DNA content smaller than that of G_0_/G_1_ (region of M1 in figure 4) are considered to be apoptotic cells. As shown in Fig. 4, 5-aza-CdR did not induce apoptosis in A549 cells at 0.1 µM (Fig. 4B) or 1 µM for 72 hrs (Fig. 4C). In addition, A549 cells were treated with 5-aza-CdR at 1 µM for 48 hrs or 72 hrsand protein was extracted for Western blotting using anti-PARP (cleavage of PARP is a hallmark of apoptosis). Fig. 4D shows that 5-aza-CdR did not induce apoptotic cell death at limited doses. These data indicate that 5-aza-CdR alone at limited doses is not able to induce apoptosis and thus the positive result in the comet assay results mainly from 5-aza-CdR induced DNA damage.

**Figure 5:**
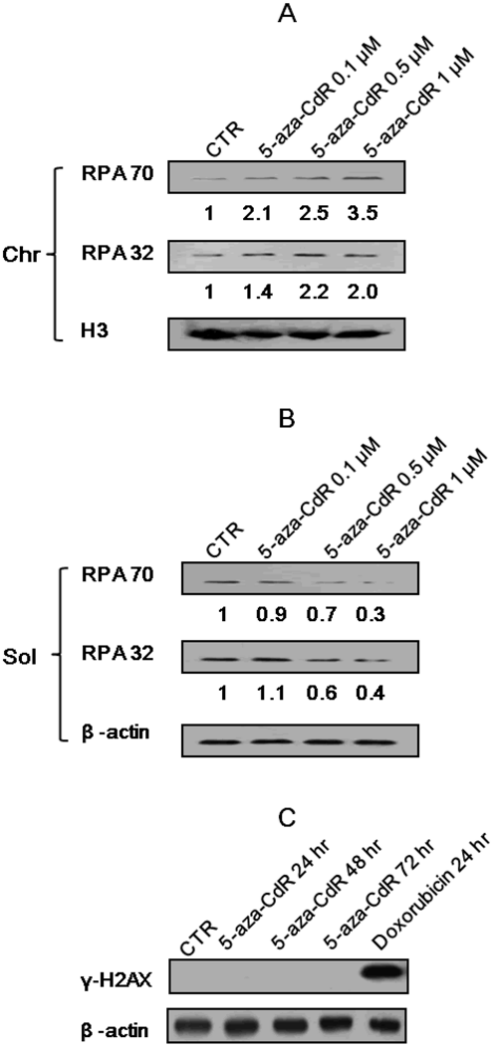
5-aza-CdR induces DNA damage with a single-strand break (SSB). (A–B) H719 cells were treated with 5-aza-CdR (0.1–1 µM) for 48 h, and cellular extracts including the chromatin fraction (A, Chr) and soluble fraction (B, Sol) were isolated for Western blotting to detect changes in RPA70 and RPA32. The band intensity of untreated sample was set as 1. The numerical value of each sample represents the percentage of band intensity relative to that of untreated sample. (C) H719 cells were also treated with 5-aza-CdR at 1 µM for 24, 48 or 72 hrs, and protein was then extracted for Western blotting using anti-γ-H2AX. Cells were treated with doxorubicin at 1 µM for 24 hrs as a positive control (far right lane).

In general, DNA damaging agents induce DNA single-strand breaks (SSB) or double-strand breaks (DSB). To determine which kind of DNA damage is induced by 5-aza-CdR, we performed Western blotting using anti-RPA (replication protein A) or γ-H2AX. Most DNA damaging agents induce cellular responses through activation of ATR (ATM and Rad3-related) [36]–[40], and the activation of ATR is largely dependent on an intermediate molecule RPA [41], [42]. Chromatin was extracted from 5-aza-CdR treated H719 cells and chromatin-bound RPA content was determined. Fig. 5A shows that chromatin-bound RPA was increased in response to increased 5-aza-CdR, indicating that 5-aza-CdR induced DNA damage may be single-strand breaks (SSB). On the other hand, γ-H2AX is considered to be a hallmark of DNA double-strand breaks (DSB) [43], and Western blotting was therefore performed using ant-γ-H2AX to rule out the possibility of 5-aza-CdR induced DSB. Fig. 5B shows that 5-aza-CdR at 1 μ did not induce DSB. However, 5-aza-CdR at higher concentration (>5 µM) induced dose-dependent DSB (data not shown). These data demonstrate that 5-aza-CdR induced DNA SSB or DSB is dosage dependent. In this study, 5-aza-CdR was almost always used at lower doses (1 µM) and thus the 5-aza-CdR induced DNA damage is SSB.

### Depsipeptide specifically inhibits removal of incorporated 5-aza-CdR and enhances nucleoside analog induced cytotoxicity

**Figure 6:**
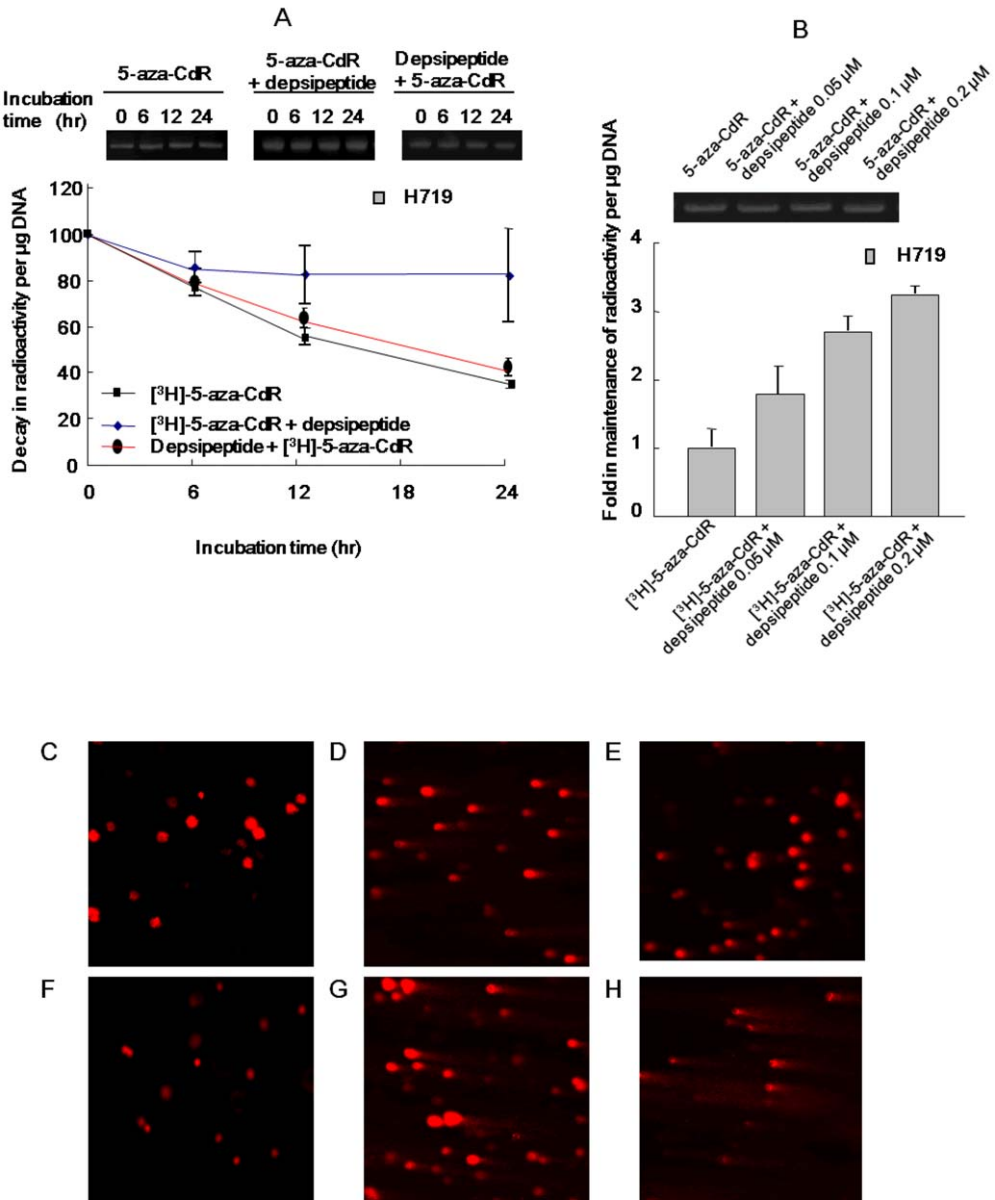
Depsipeptide inhibits removal of incorporated 5-aza-CdR from DNA. (A) H719 cells were incubated with [^3^H]-5-aza-CdR for 48 hrs, with presence or absence of depsipeptide at 0.1 µM for the first 6 hrs (depsipeptide+[^3^H]-5-aza-CdR) or last 6 hrs ([^3^H]-5-aza-CdR+depsipeptide) and then washed with cold PBS. The cells were replaced in fresh medium for further incubation for different intervals and the DNA was then extracted with cold TCA. Relative radioactivity of each sample at different time points was compared to the radioactivity of the cells at the beginning of the incubation. (B) H719 cells were treated with [^3^H]-5-aza-CdR for 48 hrs, and depsipeptide at 0.05, 0.1 and 0.2 µM was then added to the cells for the last 6 hrs. At 24 hrs after incubation, cells were harvested and radioactivity was counted. Same amount of DNA was loaded and run at 1% of agarose gel at 100 V for 5 min as loading control, which is shown at upper panel of Fig. 6A and Fig. 6B, respectively. (C–E) Comet assay shows that 5-aza-CdR induced DNA damage recovered when the treated cells were incubated in 5-aza-CdR free medium for 12 hrs. (C) Untreated control, (D) 5-aza-CdR at 1 µM for 24 hrs without incubation, and (E) 5-aza-CdR at 1 µM for 24 hrs, followed by washing for further incubation in a drug-free medium for 12 hrs. (F–H) Recovery of DNA damage induced by 5-aza-CdR was inhibited by depsipeptide treatment (0.1 µM for the final 6 hrs). (F) Depsipeptide treated cells (0.1 µM) for 6 hrs, (G) Cells were treated with 5-aza-CdR (1 µM for 24 hr) and depsipeptide (0.1 µM for last 6 hrs) without incubation, or (H) Cells treated as above and further incubated in a drug-free medium for 12 hrs.

Because DNA damage induced by low dose of 5-aza-CdR is reversible, it is particularly important to understand how HDAC inhibitor enhances 5-aza-CdR-induced toxicity. In addition, as sequential treatment of 5-aza-CdR and HDAC inhibitor is more effective in inducing cell death [44], we were led to consider whether HDACI increases uptake of 5-aza-CdR into DNA or decreases removal of incorporated 5-aza-CdR from DNA, thus enhancing 5-aza-CdR-induced cytotoxicity. First, depsipeptide (0.1 µM) and [^3^H]-5-aza-CdR (1 µM) were added together to A549 cells for an initial 6 hrs, and the cells were then continuously cultured in medium with [^3^H]-5-aza-CdR alone for another 42 hrs. Radioactivity was counted at 0–24 hrs after cells were incubated in a radioactivity-free medium. By comparing samples treated with [^3^H]-5-aza-CdR alone to samples treated with [^3^H]-5-aza-CdR plus depsipeptide, no difference in radioactivity was observed, indicating that depsipeptide does not promote incorporation of [^3^H]-5-aza-CdR into DNA (Fig. 6A). To test whether depsipeptide affects removal of incorporated 5-aza-CdR, a decay analysis of incorporated [^3^H]-5-aza-CdR was carried out in A549 cells. Cells were treated with [^3^H]-5-aza-CdR at 1 µM for 48 hrs, with and without depsipeptide at 0.1 µM for the final 6 hrs of treatment, and the cells were then washed and replaced in fresh medium (without experimental reagents) for 6, 12 and 24 hrs. Figure 6A shows that incorporated [^3^H]-5-aza-CdR gradually decreased during incubation in [^3^H]-5-aza-CdR free medium. Radioactivity per µg DNA at 6, 12 and 24 hrs in cells treated with [^3^H]-5-aza-CdR alone, for example, was decreased to 78.44±7.08%, 57.5±3.88% and 36.05±1.79% respectively, compared to the radioactivity at the beginning of incubation. This reflects removal of [^3^H]-5-aza-CdR from DNA. However, addition of depsipeptide significantly postponed the removal of [^3^H]-5-aza-CdR from DNA, and radioactivity per µg DNA at 6, 12 and 24 hrs was 80.3±13.21%, 78.71±12.72% and 82.5±20.47%, respectively, compared to the radioactivity at the beginning of incubation. This depsipeptide induced decrease in removal of [^3^H]-5-aza-CdR was also dependent on depsipeptide concentration. For example, the radioactivity of cells treated with [^3^H]-5-aza-CdR and depsipeptide at 0.05 µM, 0.1 µM and 0.2 µM showed 1.8-, 2.7- and 3.25 -fold increases respectively over that of cells treated with [^3^H]-5-aza-CdR alone (Fig. 6B). In addition, 5-aza-CdR induced DNA damage also gradually recovered when cells were cultured in a 5-aza-CdR free medium. The length and area of the DNA tail induced by 5-aza-CdR were shorter and smaller when cells were cultured in 5-aza-CdR free medium for 12 hrs as compared to the cells without drug-free incubation (Fig. 6C–E). This process of recovery from 5-aza-CdR induced DNA damage was significantly suppressed by depsipeptide, so that the length and area of the tails in the comet assay were longer and larger when cells were treated with 5-aza-CdR and depsipeptide together (Fig. 6F–H). The depsipeptide-induced suppression of recovery from 5-aza-CdR induced DNA damage is summarized in table 2. These data suggest that HDAC inhibitor may suppress recovery from 5-aza-CdR induced DNA damage and synergistically induce inhibition of cell proliferation.

**Table 2:** Recovery from 5-aza-CdR-induced DNA damage and inhibition of recovery by depsipeptide were analyzed with the comet assay

Treatment	Tail area/Total area ( % )	DNA tail length
CTR	6.6±2	2.2±0.2
Depsipeptide 6 hr	6.9±2	2.3±0.2
5-aza-CdR 24 hr	40.2±4	26.2±2.0
5-aza-CdR 24 hr+incubation 12 hr	26.6±4	9.8±1.4
5-aza-CdR 24 hr+incubation 24 hr	14.8±2	4.8±1.2
5-aza-CdR+depsipeptide 24 hr	45.4±6	27.9±3.2
5-aza-CdR+depsipeptide 24 hr+incubation 12 hr	53.4±6	40.2±3.4
5-aza-CdR+depsipeptide 24 hr+incubation 24 hr	72.2±14	70.1±10.3

A549 cells were treated with 5-aza-CdR (1 µM for 24 hrs), depsipeptide (0.1 µM for 6 hrs) alone or with both drugs in combination. The treated cells were immediately assayed with comet assay or washed and further incubated in a drug-free medium for 24 hrs and then assayed with comet assay.

### HDAC inhibitor acted selectively with nucleotide analog to induce inhibition of cell proliferation

**Figure 7:**
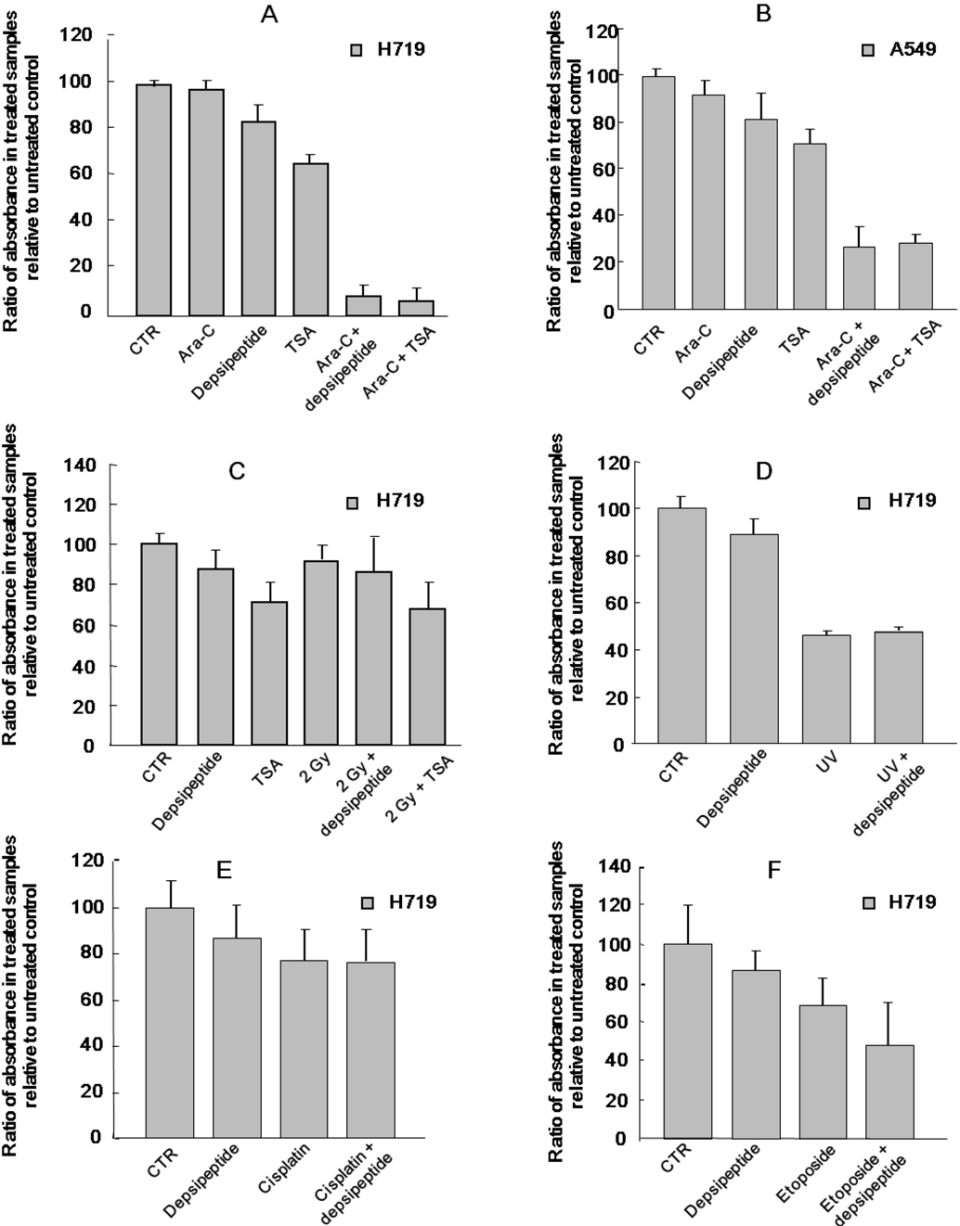
HDAC inhibitors selectively cooperated with nucleoside analog to synergistically induce inhibition of cell proliferation. (A) H719 cells or (B) A549 cells were treated with Ara-C at 0.1 µM for 72 hrs in the presence or absence of HDACI (depsipeptide 0.1 µM or TSA 2 µM for the final 6 hrs). The cells were then washed and further incubated for 24 hrs for MTT assay. (C) Effect of interaction of HDACI and irradiation at 2 Gy was determined with the MTT assay. H719 cells were irradiated at 2 Gy, or treated with depsipeptide at 0.1 µM, or TSA at 2 µM for 6 hrs alone or in combination. (D) H719 cells were also treated with UV at 128 J/m^2^, with or without depsipeptide treatment. (E) H719 cells were treated with cisplatin at 0.1 µM for 48 hrs or (F) with etoposide at 0.2 µM for 48 hrs, and then depsipeptide at 0.1 µM was separately added to the treated cells. All data above is the result of two separate experiments.

To determine whether HDACI selectively enhances NA-induced cytotoxicity, H719 cells were treated with Ara-C, another cytidine analog, and depsipeptide or TSA was then added to the cells to observe changes in cell proliferation assayed with MTT. It was of interest that both depsipeptide and TSA significantly enhanced Ara-C induced inhibition of cell proliferation (Fig. 7A–B). However, this synergistic inhibition of cell proliferation was not observed in the cells treated with depsipeptide and other DNA damage inducers such as γ-ray (Fig. 7C), UV (Fig. 7D), cisplatin (Fig. 7E) or etoposide (Fig. 7F). These data suggest that HDACI selectively acts in concert with NAs to induce inhibition of cell proliferation.

## Discussion

It is clear that 5-aza-CdR is a potent demethylating agent, in that 5-aza-CdR is readily incorporated into DNA, and the nitrogen substitution for the 5-carbon in the cytosine moiety is able to confer the ability to trap DNA methyltransferase 1 [16], [28], [45]. In theory, inhibition of both DNA methylation and HDAC will increase expression of genes if they are methylated in their promoter regions, because methyl-binding proteins specifically bind to methylated DNA and recruit HDAC activity [11], [46]. In fact, the effectiveness of this combination in treatment of cancer is the subject of a number of human clinical trials by several different groups [7], [8], [47], [48]. There are reports indicating that methylation in the promoter region of caspase 8 or caspase 1 is one reason that cells develop resistance to chemotherapy [49], [50], and treatment with 5-aza-CdR could help abolish this resistance and restore chemotherapy induced cell death [49], [51]. However, in most cases of 5-aza-CdR induced apoptosis, it is generally unclear which gene or pathway leads to this effect. We tested the methylation status of several important apoptotic related genes including *p53, bcl-2, bax* and *caspase 8*, but all of these were hypomethylated in the cells tested (data not shown). In addition, in the present study, although DNMT1/DNMT3A/DNMT3B were effectively knocked down (Fig. 1A–C), depsipeptide was not able to induce a synergistic inhibition of cell proliferation (Fig. 2A). Therefore, the synergistic effects of 5-aza-CdR in combination with HDACI in inhibition of cell proliferation most likely have an alternative mechanism that is methylation-independent.

In addition to its demethylating function, 5-aza-CdR also has anti-neoplastic activity which operates by damaging DNA [24], [25], [52]. Most NAs exert their cytotoxicity through interference with DNA replication. It has been found experimentally that NAs incorporated into DNA and abases inhibit template function and chain elongation [26]. In addition, the incorporated abases competitively inhibit DNA polymerase α in opposition to the normal substrate deoxycytidine 5′-triphosphate [53]. The NAs also cause an unusual reiteration of DNA segments, which results in multiple copies of limited portions of DNA and increases the possibility of recombination, crossover, gaps and breaks [54]. 5-aza-CdR differs from physiological cytidine by the presence of a nitrogen at the 5- position of the heterocyclic ring and this substitution renders the ring chemically unstable and leads to spontaneous decomposition of its compounds in neutral solution [26]. The incorporated 5-aza-CdR or its unstable form which has a ring that breaks open inducing DNA damage, consequently may elicit many different cellular responses using different pathways to repair the resultant DNA damage. Consistent with these reports, our data demonstrate that 5-aza-CdR can induce DNA damage in a dose-dependent manner (Fig. 3C–E), and the degree of DNA damage induced by 5-aza-CdR also parallels the amount of incorporated [3H]-5-aza-CdR (Fig. 3A–B compares to Fig. 3C–E). Here it should be pointed out that 5-aza-CdR induced DNA damage is a SSB at lower doses as demonstrated by an increase in chromatin-bound RPA, and absence of formation of γ-H2AX (Fig. 5A–B). However, when 5-aza-CdR was used at a higher concentration (for example, >5 µM), formation of γ-H2AX was observed in response to 5-aza-CdR treatment (data not shown). We propose that 5-aza-CdR is randomly incorporated into DNA as a cytosine substitution, and thus higher concentrations of 5-aza-CdR result in greater incorporation of 5-aza-CdR into DNA. On the other hand, as single-strand breaks can be turn into DSBs if the replication fork is interrupted [55], [56], the possibility of co-existence of SSB and DSB is reasonable, and it may be dependent of dosage and degree of DNA damage.

However, cells have a capacity to exclude harmful abases and repair DNA damage induced by different stimuli. A chief difference from radiation- or other stimuli-induced DNA damage is that NA induced DNA damage usually is limited to an alteration in a DNA base or a nucleotide. The enzymes that are responsible for excision of incorporated 5-aza-CdR or Ara-C have not been clearly identified. However, more than 10 distinct DNA glycosylases have been found in cells which can repair abase-induced DNA damage, including enzymes specific for excision of uracil or 5-methyl-uracil [57]. There are two classes of enzymes for base excision repair: “short patch” involved enzymes and “long patch” involved enzymes [58]. The base excision repair (BER) initiated by enzymes for the “short patch” are glycosylases that include UNG, TDG, SMUG1 and MBD4. For example, uracil represents an endogenous DNA lesion that results from deamination of cytosine or incorporation of uracil instead of T during replication [59]. As a uracil DNA glycosylase, UNG, has been considered to be a key enzyme for removal of uracil in DNA [60]. However, although UNG is highly selective for uracil in DNA, it also removes certain bases that are structurally related to cytosine. These related bases include 5-fluorouracil in DNA [61], [62], 5-hydroxy-2′-deoxyuridine [63], [64] and alloxan [64]. In *E. coli*, for example, mutation of uracil DNA glycosylase leads to a 50-fold increase of 5-fluorouracil incorporated into DNA [65]. In human lymphoblasts, UNG was also found to remove 5-fluorouracil in vitro [61]. As early as 1980s, it was reported that 5-aza-CdR induced DNA damage results from a single-strand break and glycosylase recognizes and removes 5-aza-CdR from DNA [66]. In this study, we analyzed expression of enzymes that are mainly responsible for BER in depsipeptide treated cells, and only UNG expression was obviously inhibited (data not shown). However, UNG may be not directly involved in excising the 5-aza-CdR adduct since RNAi against UNG is not sufficient to mimic the effect of depsipeptide in its synergistic effect in inhibiting cell proliferation with 5-aza-CdR (data not shown). We also tested to see whether depsipeptide influences activities of enzymes for “long patch repair” including human apurinic/apyrimidinic endonuclease 1 (APE1) and flap endonuclease 1 (FEN1). However, depsipeptide did not inhibit expression of APE1 and FEN1 (data not shown). These data reflect the complexity of 5-aza-CdR induced DNA damage and the enzymes involved in repairing the damaged DNA.

5-aza-CdR-induced DNA damage was previously reported to come from a bulky adduct of 5-aza-CdR and DNMT1 [16]. This adduct may be excised by several enzymes that are involved in nucleotide excision repair (NER) [58]. Therefore, identification of enzymes which operate in NER is a critical step for the future.

HDAC inhibitors seem likely to be specific in their synergy with NAs to induce inhibition of cell proliferation, since no inhibition of cell proliferation after treatment with depsipeptide in cells irradiated with γ-rays or treated with other DNA damage inducers was observed (Fig. 7C–F). In support of this, HDAC inhibitor MS-275 interacts synergistically with different nucleoside analogs, but not with other DNA damaging agents to induce apoptosis in different human cancer cells [48]. These data suggest that HDACI may specifically suppress expression or activity of enzymes that are involved in repairing nucleoside analog-induced DNA damage. Therefore, to identify which enzyme(s) is responsible for repairing NA incorporation, understanding of cytosine analog-induced DNA damage is extremely critical and may be useful for designing new therapeutic strategies to treat cancer.

In conclusion, in this study we demonstrate that 5-aza-CdR exerts cytotoxicity by incorporating into DNA and inducing DNA damage. HDACI may interfere with the repair process of nucleoside analog induced DNA damage, and thus lead to a significant inhibition of cell proliferation. We believe that this novel finding will be helpful in formulating a new strategy to treat cancer more effectively in the clinic.
